# New Tribe of Aborgines, without Hair

**Published:** 1859-03

**Authors:** 


*New Tribe of Aborigines, without Hair.*—The discovery of a new tribe of aborigines is thus reported in the Sidney Empire:

"A gentleman, who, in May last, was at a remote station down the Balonne, called Gooee, about 100 miles below Surat, fell in with four blacks, who had come to that part of the Balonne a few days previous, and who appeared to belong to a tribe unknown to white men. They presented the remarkable peculiarity of being entirely without hair, and they stated that neither the males nor females of their tribes had hair on their bodies at any period of life. The complete baldness gave them a strange unearthly appearance, at which it is said the Balonne blacks were at first very much terrified. These aboriginal strangers said they saw white men's bones and equipments beyond the river Barrow or Warrego, from which they had come. It is conjectured that these remains may be those of Leichardt and his party, and we believe the whole particulars have been communicated to the government, with the view of a fresh search being made to clear up the mystery of the long-missing travelers."—*London Lancet*, from *Boston Med. and Surg. Journal.*

